# Georgia State Sanitarium

**Published:** 1901-12

**Authors:** 


### GEORGIA STATE SANITARIUM.

WE have received a copy of the annual report of the Board of Trustees of the Georgia State Sanitarium for the fiscal year ending September 1, 1901.

The number of inmates on September 1, 1901, was 2,595. Of this number 1,822 are whites, 773 colored. During the past year 451 whites and 252 colored were admitted. During the same period the number of inmates who were discharged, eloped, removed or died, was 651. The average number of inmates during the year, was 2,573, as against 2,495 last year; that is 78 more. The percentage of recoveries, based on the number received during the year, was 46.9, as against 49.$\frac{3}{4}$ for the previous year. The general health of the inmates, considering the overcrowding, was good. Tuberculosis made its appearance, but small-pox was successfully evaded, owing to enforcement of the rule forbidding reception of patients from counties in which small-pox exists. Two new buildings are under construction, which when completed will cost $150,000 (the amount appropriated by the General Assembly for this purpose), and will accommodate 1,448 patients. This, with the purposed scheme for colonizing colored males, will relieve the overcrowding and afford accommodation for all the insane in the State for at least ten years to come. It is shown by the superintendent's report on expenditures, that the per capita cost of maintenance of the Sanitarium is 30.29 cents per diem, which is slightly in excess of the cost for the previous year. With this exception, the present cost is the lowest in the history of the institution.

A great deal is done to occupy and divert the inmates. Such patients as are fit, are put to easy tasks and much is done for the amusement of the unfortunates. This has been found to be an important adjuvant to medical treatment and to materially help on to recovery.

It appears from the report of the Pathologist, Dr. Perry, (to whom we tender thanks for the courtesy of sending us the report), that the principal lesions found post mortem were concerned with the kidneys, heart and arterial system. Dr. Perry has been devoting considerable attention to the examination of the urine with a view of determining the role played in deficient renal elimination as a causative factor of mental disease.

The training school for nurses is being successfully conducted under the supervision of Miss Cross.

The superintendent makes an earnest appeal for the segregation of the convicts, and it is earnestly hoped that his recommendations will be carried out, and that the non-criminal insane will not have added to their afflictions the necessity of foregathering with convicts.

The report is neatly printed and illustrated with half-tone reproductions of photographs of the buildings. Errors in proof-reading are many and somewhat mar the report.

---

DR. J. F. ALEXANDER, one of the oldest and best known physicians of Atlanta, died November 20th, aged seventy-seven years.

---

THE evidences of approaching famine in many of the governments of Russia are, according to the *Medical Record*, accumulating, and the officials are beginning already to take measures to reduce to a minimum the inevitable suffering that will ensue. The sum of 96,000 rubles ($48,000) has been appropriated for the government of Saratoff; 407,000 rubles ($203,000) for Tauris ; 100,000 rubles ($50,000) for the non-military peasants in the Don basin, and 190,000 rubles ($95,000), with a supply of autumn seed, for the government of Yekaterinslaff. On August 15 the central government's famine fund amounted to only 530,000 rubles ($265,000). Emperor Nicholas ordered that this be increased to 14,000,000 rubles ($7,000,000). According to the reports of the governors, State assistance is required in nineteen provinces, not counting the country of the Don Cossacks.

